# Modelling of Flexible Adhesives in Simple Mechanical States with the Use of the Darijani–Naghdabadi Strain Tensors and Kirchhoff–de Saint-Venant Elastic Potential

**DOI:** 10.3390/polym13101639

**Published:** 2021-05-18

**Authors:** Paweł Szeptyński, Matija Gams, Arkadiusz Kwiecień

**Affiliations:** 1 Division of Structural Mechanics and Material Mechanics, Faculty of Civil Engineering, Cracow University of Technology, 31-155 Cracow, Poland; akwiecie@pk.edu.pl; 2 Chair of Structural and Earthquake Engineering, Department of Civil Engineering, Faculty of Civil and Geodetic Engineering, University of Ljubljana, 1000 Ljubljana, Slovenia; matija.gams@fgg.uni-lj.si

**Keywords:** generalized measures of strain, nonlinear elasticity, flexible adhesives

## Abstract

Practical aspects of modelling of flexible adhesives with the energy conjugate measures of stress and strain of the Darijani–Naghdabadi (D-N) family are discussed. A possibility of description of materials exhibiting non-linear physical characteristics with the use of non-linear geometric relationships and linear elastic constitutive law is considered. Nominal stress vs. stretch relations are specified in cases of simple tension and simple shear with the use of the Kirchhoff–de Saint-Venant elastic potential and D-N energy conjugate stress and strain measures. Obtained theoretical estimates were compared with experimental results of simple tension and simple shear tests performed on Sika PM polyurethane (Cracow, Sika Poland). The deformation rate was fixed in order to minimize the influence of viscosity. Values of parameters α,β in the definition of the D-N strain tensor were optimized in order to provide good agreement between model predictions and experimental results. Observed discrepancies indicate that the proposed approach is not appropriate for constitutive modelling of the PM polymer. The presented approach is proposed to be used as a simple design model providing practical formulas describing the behavior of materials of non-linear characteristics in chosen mechanical states. Admissible values of exponents α,β are discussed regarding its bijectivity in a limited range of variation of principal stretches.

## 1. Introduction

Flexibility of joints based on adhesives of relatively high compliance compared to other similar solutions (e.g., flexible polyurethane resin and stiffer epoxy resin) enables increases in the total bearing capacity of certain composite structural elements, which is, however, accompanied by lower overall stiffness of the system. Various practical aspects of the application of stiff and flexible joints and adhesives were discussed in [1,2,3,4,5,6,7]. Flexible joints are used in forms of PUFJ (polyurethane flexible joints) and FRPU (fiber-reinforced polyurethane). These structural connectors can be used as repair, strengthening or protection solutions in RC (reinforced concrete) and masonry structures, because they are capable of transferring high loads and large deformations [8]. They are constructed from special polyurethanes of non-linear and visco-elastic characteristic; thus, different material properties can be expected in cases of various strain rates [9] and various environmental conditions [10]. Model descriptions of PUFJ and FRPU are needed for calculations when practical engineering applications in buildings are considered. Experimental tests provide evidence that failure mechanisms in flexible joints are mainly related to tensile and shear stresses present in adhesives. 

Adhesives are considered generally physically non-linear materials. For the description of such materials, appropriate mathematical modelling is required [11]. For flexible adhesives, physical non-linearity becomes even more important, because for any given load, the interval of the strain accounted for is larger than in the case of more stiff materials. Constitutive laws which are commonly used in order to model polymer materials are, e.g., the Neo-Hookean solid model, models by Mooney and Rivlin [12], Ogden [13], Yeoh [14], etc. An alternative approach was proposed in [15,16,17]—instead of formulating a non-linear constitutive law binding chosen stress and strain tensorial measures, a linear constitutive law is formulated for a different set of stress and strain tensors. In particular, the use of the Darijani–Naghdabadi strain tensor family [15], which may be considered a generalization of the Seth–Hill strain tensor family [18,19], emerged as an efficient solution that enables accounting for non-linear characters of constitutive relationships.

## 2. Darijani–Naghdabadi Measures of Strain

Darijani and Naghdabadi have proposed in [15] the following approach to define a new family of tensorial strain measures. We consider the material deformation gradient F such that detF>0. According to the polar decomposition theorem, F can be represented in the form of product:

(1) F=R U=V R

where R is the rotation tensor and U and V are the right and left stretch tensor, respectively. Let us denote the ith eigenvector of U with $u_{i}$ and corresponding eigenvalue (principal stretch) with $\lambda_{i}$. Then

(2) $$U=\overset{3}{\underset{i=1}{\sum }}\lambda_{i}u_{i}⊗u_{i}$$

A generalized measure of strain in material description which is coaxial with the right stretch tensor may be defined as a tensor-valued function of tensor argument in the following way:

(3) $$E^{f}\left(U\right)=\overset{3}{\underset{i=1}{\sum }}f\left(\lambda_{i}\right)u_{i}⊗u_{i}$$

where $f\left(\lambda_{i}\right)$ is a strictly increasing scalar function defined for non-negative values of its argument, namely:

$$\forall_{a,b\in \left({\mathbb{R}}_{+}\cup \left\{0\right\}\right)}\;\left[b>a\right]\;\Rightarrow \left[f\left(b\right)>f\left(a\right)\right]$$

satisfying the following constraints:

$$\begin{array}{cccc} f\left(\lambda =1\right)=0, & {\frac{df}{d\lambda }|}_{\lambda =1}=1, & \underset{\lambda \to 0}{\lim}f\left(\lambda \right)=-\infty , & \underset{\lambda \to +\infty }{\lim}f\left(\lambda \right)=+\infty \end{array}$$

It can be checked that all the above requirements are met by a function given by a linear combination of power functions of the following form:

(4) $$f\left(\lambda \right)=\frac{1}{\alpha +\beta }\left(\lambda^{\alpha }-\lambda^{-\beta }\right),\;\;\;\mathrm{where}\;\alpha \beta >0\;\;\;\mathrm{or}\;\{\begin{array}{c} \alpha \to 0\\ \beta \to 0 \end{array}$$

If one of the exponents is equal to 0, we obtain the Seth–Hill strain tensors family:

- Green–de Saint-Venant strain tensor

$$\alpha =2,\beta =0\;\mathrm{or}\;\alpha =0,\beta =-2\;\Rightarrow E^{\left(2,0\right)}=\frac{1}{2}\left(U^{2}-1\right)$$

- Biot strain tensor

$$\alpha =1,\beta =0\;\mathrm{or}\;\alpha =0,\beta =-1\;\Rightarrow E^{\left(1,0\right)}=\left(U-1\right)$$

- Hencky strain tensor

$$\alpha \to 0,\beta =0\;\mathrm{or}\;\alpha =0,\beta \to 0\;\Rightarrow E^{\left(0,0\right)}=\ln\left(U\right)$$

- Almansi strain tensor

$$\alpha =-2,\beta =0\;\mathrm{or}\;\alpha =0,\beta =2\;\Rightarrow E^{\left(0,2\right)}=\frac{1}{2}\left(1-U^{-2}\right)$$

It must be noted, however, that in all of the above cases (except for α,β→0), the requirement of an infinite value of a strain measure in a limited case strain (infinite stretch λ→∞, infinite compression λ→0) is not satisfied. Indeed, these are the power functions of non-zero exponents that provide infinite values: negative for λ→0 if the exponent is negative, and positive for λ→∞ if the exponent is positive. Lacking one of those components yields in violating one of the limit case requirements—it has no physical significance, but it is only a mathematical feature of a measure chosen for the description of deformation.

## 3. Energy Conjugate Stress Measures for the DN Strain Tensors

For a given measure of strain E, an energy conjugate measure of stress T is defined as the one for which:

(5) $${∭}_{V}\left(T_{ij}{\dot{E}}_{ij}\right)dV={∭}_{V}\left(t_{ij}d_{ij}\right)dV=P_{s}$$

where $P_{s}$ is the power of elastic strain, $t_{ij}$ are the components of the Cauchy stress tensor (true stress tensor), and

$$d_{ij}=\frac{1}{2}\left({\dot{u}}_{i,j}+{\dot{u}}_{j,i}\right)$$

are the stretch rate tensor components. Examples of pairs of the energy conjugate stress and strain tensors are:

- Cauchy stress tensor and linear part of the Almansi–Hamel strain tensor (symmetric part of the spatial displacement gradient)

$$T_{\sigma },\;\;\eta =\frac{1}{2}\left[\nabla_{x}u+{\left(\nabla_{x}u\right)}^{T}\right]$$

- Kirchhoff stress tensor and linear part of the Almansi–Hamel strain tensor (symmetric part of the spatial displacement gradient)

$$T_{\tau }=JT_{\sigma },\;\;\eta =\frac{1}{2}\left[\nabla_{x}u+{\left(\nabla_{x}u\right)}^{T}\right]$$

- Piola–Kirchhoff stress tensor of the first kind and material deformation gradient

$$T_{R}=JT_{\sigma }F^{-T}\;,\;\;F=x⊗\nabla_{X}$$

- Piola–Kirchhoff stress tensor of the second kind and Green–de Saint-Venant strain tensor

$$T_{S}=JF^{-1}T_{\sigma }F^{-T}=T^{\left(2,0\right)}\;,\;\;E^{\left(2,0\right)}=\frac{1}{2}\left(U^{2}-1\right)$$

- Jaumann stress tensor and Biot strain tensor

$$T_{J}=\frac{1}{2}\left(T_{S}U+UT_{S}\right)=T^{\left(1,0\right)},\;\;E^{\left(1,0\right)}=\left(U-1\right)$$

It is worth noting that $\dot{\eta }=d$. We are looking for stress tensors $T^{\left(\alpha ,\beta \right)}$ which are energy conjugates to the Darijani–Naghdabadi strain tensors $E^{\left(\alpha ,\beta \right)}$, namely, for which:

(6) $${∭}_{V}\left(T_{ij}^{\left(\alpha ,\beta \right)}{\dot{E}}_{ij}^{\left(\alpha ,\beta \right)}\right)dV=P_{s}$$

It was shown in [15,20] that with the use of the Hill’s principal axis method and with the notion of energy conjugacy, the relationship between components of two stress tensors $T^{\left(\alpha ,\beta \right)}$ and $T^{\left(\vartheta ,\psi \right)}$ in the basis of eigenvectors of the right stretch tensor may be expressed as follows:

(7) $$\left\{\begin{array}{c} T_{ij}^{\left(\alpha ,\beta \right)}=\frac{\left(\alpha +\beta \right)}{\left(\vartheta +\psi \right)}\begin{array}{cc} \frac{\vartheta \lambda_{i}^{\vartheta -1}+\psi \lambda_{i}^{-\psi -1}}{\alpha \lambda_{i}^{\alpha -1}+\beta \lambda_{i}^{-\beta -1}}T_{ij}^{\left(\vartheta ,\psi \right)} & \mathrm{for}\;\left(i=j\right)\;\mathrm{or}\;\left(\lambda_{i}=\lambda_{j}\right) \end{array}\\ \begin{array}{cc} T_{ij}^{\left(\alpha ,\beta \right)}=\frac{\left(\alpha +\beta \right)}{\left(\vartheta +\psi \right)}\frac{\left(\lambda_{i}^{\vartheta }-\lambda_{i}^{-\psi }\right)-\left(\lambda_{j}^{\vartheta }-\lambda_{j}^{-\psi }\right)}{\left(\lambda_{i}^{\alpha }-\lambda_{i}^{-\beta }\right)-\left(\lambda_{j}^{\alpha }-\lambda_{j}^{-\beta }\right)}T_{ij}^{\left(\vartheta ,\psi \right)} & \mathrm{for}\;\left(i\neq j\right)\;\mathrm{and}\;\left(\lambda_{i}\neq \lambda_{j}\right) \end{array} \end{array}\right.$$

In this case, when α→0,β→0, we obtain:

(8) $$\left\{\begin{array}{c} T_{ij}^{\left(\alpha ,\beta \right)}=\begin{array}{cc} \frac{\lambda_{i}\left(\vartheta \lambda_{i}^{\vartheta -1}+\psi \lambda_{i}^{-\psi -1}\right)}{\left(\vartheta +\delta \right)}T_{ij}^{\left(\vartheta ,\psi \right)} & \mathrm{for}\;\left(i=j\right)\;\mathrm{or}\;\left(\lambda_{i}=\lambda_{j}\right) \end{array}\\ \begin{array}{cc} T_{ij}^{\left(\alpha ,\beta \right)}=\frac{\left(\lambda_{i}^{\vartheta }-\lambda_{i}^{-\psi }\right)-\left(\lambda_{j}^{\vartheta }-\lambda_{j}^{-\psi }\right)}{\left(\vartheta +\psi \right)\left[\ln\lambda_{i}-\ln\lambda_{j}\right]}T_{ij}^{\left(\vartheta ,\psi \right)} & \mathrm{for}\;\left(i\neq j\right)\;\mathrm{and}\;\left(\lambda_{i}\neq \lambda_{j}\right) \end{array} \end{array}\right.$$

Additional relations enabling the expression of tensor $T^{\left(\alpha ,\beta \right)}$ in terms of the Cauchy stress tensor $T_{\sigma }$ were also found:

(9) $$T^{\left(1,1\right)}+U^{-1}T^{\left(1,1\right)}U^{-1}=U^{-1}R^{T}T_{\sigma }R+R^{T}T_{\sigma }RU^{-1}$$

(10) $$T_{\sigma }=\frac{1}{2J}\left[F\;T^{\left(2,2\right)}F^{T}+F^{-T}T^{\left(2,2\right)}F^{-1}\right]$$

(11) $$T_{\sigma }=\frac{1}{J}\left[F\;T^{\left(2,0\right)}F^{T}\right],\;\;T^{\left(1,0\right)}=\frac{1}{2}\left[T^{\left(2,0\right)}U+UT^{\left(2,0\right)}\right]$$

(12) $$T_{\sigma }=J^{-1}\left[F^{-T}\;T^{\left(-2,0\right)}F^{-1}\right],\;\;T^{\left(-1,0\right)}=\frac{1}{2}\left[T^{\left(-2\right)}U^{-1}+U^{-1}T^{\left(-2,0\right)}\right]$$

Equations (7)–(12) enable us to find energy conjugate stress tensors for chosen strain tensors of the DN family.

## 4. Kirchhoff–de Saint Venant Potential in Description of Flexible Adhesives

The form of the elastic potential W(E) relating the components of chosen stress tensor $T_{ij}$ and chosen strain tensor $E_{ij}$ with the relation

(13) $$T_{ij}=\frac{\partial W}{\partial E_{ij}}$$

will generally be different if distinct pair of stress and strain measures will be chosen. In particular, if we chose the so-called Kirchhoff–de Saint-Venant potential in the form of homogeneous quadratic form of strain components

(14) $$W=\mu \;E_{ij}E_{ij}+\frac{\Lambda }{2}{\left(E_{kk}\right)}^{2}$$

yielding a linear constitutive relation for a chosen pair of stress and strain measures

(15) $$T_{ij}=2\mu \;E_{ij}+\Lambda E_{kk}\delta_{ij}$$

the constitutive relationship between any other measures of stress and strain will generally be non-linear. This observation indicates a possibility to use the linear constitutive relationship in the description of materials which are considered physically non-linear. 

Physical non-linearity is thus not solely a property of a material, but it is a feature of the model used, chosen theory, and measures of stress and strain. Indeed, the significance of non-linearity is due to its violation of linearity. A well-developed linear theory of elasticity is based on linear approximations of stress increment (equilibrium equations), strain measure being a linear combination of displacement gradient and its transpose (small strain tensor, geometrical linearity) and linear constitutive relation (Hooke’s law). A non-linear material that which within the linear theory or in terms of a priori assumed strain and stress measures (e.g., engineering strain and nominal stress, etc.) exhibits a non-linear constitutive relationship. Hence, such a material may be described either with the use of certain non-linear constitutive models within a linear theory or with the use of a different constitutive model within non-linear theory. It may emerge that a linear constitutive law may describe the material’s behavior in a sufficiently precise way for a certain measure of strain. This concept was used in [16,17] in order to describe flexible adhesives with the use of the Darijani–Naghdabadi strain measures by a proper choice of exponents α and β.

It must be emphasized, however, that this is still a description accounting only for elasticity, neglecting, e.g., viscous properties (i.e., dependency between stiffness and strain rate), which are known to be exhibited by flexible adhesives. Such an approach is thus applicable only for quasi-static processes, or for processes of fixed deformation rate [9]. For distinct strain rates, the same approach may be used, although different models (i.e., appropriate values of α,β) must be used. It is clear also that parameters α and β by no means could be considered any kind of “material constants”— rather, they are parameters of a model (in the sense of a theory as a whole). The mentioned approach still may be a useful tool in the practical description of materials of non-linear characteristics, i.e., flexible adhesives, especially in simple engineering problems, such as finding the bond length of a strengthening films.

Even though a linear constitutive law may be—at least formally—formulated in terms of any stress and strain measures, choosing only the energy conjugate pairs is justified by their specific properties, i.a., satisfying the principle of virtual velocities.

## 5. Force-Stretch Relations

### 5.1. Simple Tension

In cased of simple tension, we have:

$$\{\begin{array}{l} x_{1}=\lambda_{1}X_{1}\\ x_{2}=\lambda_{2}X_{2}\\ x_{3}=\lambda_{2}X_{3} \end{array}\;\;\Rightarrow \;\;F=\left[\begin{array}{ccc} \lambda_{1} & 0 & 0\\ 0 & \lambda_{2} & 0\\ 0 & 0 & \lambda_{3}=\lambda_{2} \end{array}\right]=U,\;\;R=1$$

True stress tensor is assumed to be of the form:

(16) $$T_{\sigma }=\left[\begin{array}{ccc} \frac{P\left(\lambda_{1}\right)}{A\left(\lambda_{1}\right)} & 0 & 0\\ & 0 & 0\\ \mathrm{sym} & & 0 \end{array}\right]$$

As a result, the Piola–Kirchhoff stress tensors are as follows:

(17) $$T_{R}=JT_{\sigma }F^{-T}=\left[\begin{array}{ccc} \frac{P\left(\lambda_{1}\right)}{A_{0}} & 0 & 0\\ 0 & 0 & 0\\ 0 & 0 & 0 \end{array}\right]=\left[\begin{array}{ccc} \frac{P\left(\lambda_{1}\right)}{A\left(\lambda_{1}\right)}\lambda_{2}^{2} & 0 & 0\\ 0 & 0 & 0\\ 0 & 0 & 0 \end{array}\right]$$

(18) $$T_{S}=JF^{-1}T_{\sigma }F^{-T}=\left[\begin{array}{ccc} \frac{P\left(\lambda_{1}\right)}{A\left(\lambda_{1}\right)}\frac{\lambda_{2}^{2}}{\lambda_{1}} & 0 & 0\\ & 0 & 0\\ \mathrm{sym} & & 0 \end{array}\right]=\left[\begin{array}{ccc} \frac{P\left(\lambda_{1}\right)}{A_{0}}\frac{1}{\lambda_{1}} & 0 & 0\\ & 0 & 0\\ \mathrm{sym} & & 0 \end{array}\right]$$

The strain tensor $E^{\left(\alpha ,\beta \right)}$ is represented as follows:

(19) $$E^{\left(\alpha ,\beta \right)}=\frac{1}{\alpha +\beta }\left[\begin{array}{ccc} \left(\lambda_{1}^{\alpha }-\lambda_{1}^{-\beta }\right) & 0 & 0\\ 0 & \left(\lambda_{2}^{\alpha }-\lambda_{2}^{-\beta }\right) & 0\\ 0 & 0 & \left(\lambda_{2}^{\alpha }-\lambda_{2}^{-\beta }\right) \end{array}\right]$$

Let us relate the energy conjugate stress tensor $T^{\left(\alpha ,\beta \right)}$ to the Piola–Kirchhoff stress tensor of the second kind $T_{S}=T^{\left(2,0\right)}$, according to Equation (8), which simplifies to the form:

(20) $$\{\begin{array}{l} T_{ij}^{\left(\alpha ,\beta \right)}=\begin{array}{cc} \frac{\left(\alpha +\beta \right)\lambda_{i}\;}{\alpha \lambda_{i}^{\alpha -1}+\beta \lambda_{i}^{-\beta -1}}S_{ij} & \mathrm{for}\;\left(i=j\right)\;\mathrm{or}\;\left(\lambda_{i}=\lambda_{j}\right) \end{array}\\ \begin{array}{cc} T_{ij}^{\left(\alpha ,\beta \right)}=\frac{\left(\alpha +\beta \right)\left(\lambda_{i}^{2}-\lambda_{j}^{2}\right)}{2\left[\left(\lambda_{i}^{\alpha }-\lambda_{i}^{-\beta }\right)-\left(\lambda_{j}^{\alpha }-\lambda_{j}^{-\beta }\right)\right]}S_{ij} & \mathrm{for}\;\left(i\neq j\right)\;\mathrm{and}\;\left(\lambda_{i}\neq \lambda_{j}\right) \end{array} \end{array}$$

This gives us:

(21) $$T_{11}^{\left(\alpha ,\beta \right)}\left(\lambda_{1}\right)=\left[\frac{P\left(\lambda_{1}\right)}{A_{0}}\right]\frac{\alpha +\beta }{\alpha \lambda_{1}^{\alpha -1}+\beta \lambda_{1}^{-\beta -1}}$$

and all other components equal to zero. From the form of the constitutive relation (Equation (15)), we conclude that the stress and strain tensors may be related with the use of the Kirchhoff–de Saint-Venant potential if, and only if, they are coaxial. It is clear that in the case of uniaxial stress state, all tensors $T^{\left(\alpha ,\beta \right)}$ and $E^{\left(\alpha ,\beta \right)}$ are coaxial; therefore, the constitutive law in the form of Equation (15) may be formulated for any chosen energy conjugate pair $T^{\left(\alpha ,\beta \right)}$ and $E^{\left(\alpha ,\beta \right)}$:

(22) $$T_{11}^{\left(\alpha ,\beta \right)}=2\mu E_{11}^{\left(\alpha ,\beta \right)}+\Lambda \left(E_{11}^{\left(\alpha ,\beta \right)}+E_{22}^{\left(\alpha ,\beta \right)}+E_{33}^{\left(\alpha ,\beta \right)}\right)$$

(23) $$T_{22}^{\left(\alpha ,\beta \right)}=T_{33}^{\left(\alpha ,\beta \right)}=2\mu E_{22}^{\left(\alpha ,\beta \right)}+\Lambda \left(E_{11}^{\left(\alpha ,\beta \right)}+E_{22}^{\left(\alpha ,\beta \right)}+E_{33}^{\left(\alpha ,\beta \right)}\right)=0$$

The latter equation relates the principal stretches in the uniaxial stress state, namely:

(24) $$\left(\lambda_{2}^{\alpha }-\lambda_{2}^{-\beta }\right)=-\nu \left(\lambda_{1}^{\alpha }-\lambda_{1}^{-\beta }\right),\;\mathrm{where}\;\;\nu =\frac{\Lambda }{2\left(\mu +\Lambda \right)}$$

After substituting Equations (21) and (24) into (22), we obtain:

(25) $$\left[\frac{P}{A_{0}}\right]=E\frac{\alpha \lambda_{1}^{\alpha -1}+\beta \lambda_{1}^{-\beta -1}}{{\left(\alpha +\beta \right)}^{2}}\left(\lambda_{1}^{\alpha }-\lambda_{1}^{-\beta }\right),\;\mathrm{where}\;\;E=\frac{\mu \left(3\;\Lambda +2\;\mu \right)}{\mu +\Lambda }$$

Equation (25) constitutes an explicit non-linear relationship between nominal stress and principal stretch, which, in turn, may be expressed in terms of absolute elongation ΔL and gauge length $L_{0}$:

$$\lambda_{1}=\frac{L_{0}+\Delta L}{L_{0}}$$

Equation (25) depends on a single constant parameter E, although the validity of this model must be verified, namely, one must find such values of α, β for which the theoretical description of the material with the use of the Kirchhoff–de Saint-Venant potential is in good agreement with experimental results. Parameter E is in fact the initial tangent Young’s modulus. If we express the obtained formula in terms of the relative elongation $\varepsilon_{1}=1+\lambda_{1}$, it can be shown that

(26) $$E_{t,0}=\frac{d}{d\varepsilon_{1}}{\left[\frac{P}{A_{0}}\right]|}_{\varepsilon_{1}=0}=\frac{d}{d\lambda_{1}}{\left[\frac{P}{A_{0}}\right]|}_{\lambda_{1}=1}=E$$

Polymer adhesives are often considered incompressible solids, such that there is no local relative volume change. In cases of uniaxial stress state:

$$\frac{dV}{dV_{0}}=J=\lambda_{1}\lambda_{2}\lambda_{3}=1\;\;\;\Rightarrow \;\;\;\;\lambda_{2}=\lambda_{3}=\lambda_{1}^{-\frac{1}{2}}$$

However, the form of the force–stretch relationship remains the same. For the parameter ν, we may then write:

$$\underset{\lambda_{1}\to 1}{\lim}\nu =\underset{\lambda_{1}\to 1}{\lim}-\frac{\left(\lambda_{2}^{\alpha }-\lambda_{2}^{-\beta }\right)}{\left(\lambda_{1}^{\alpha }-\lambda_{1}^{-\beta }\right)}=\underset{\lambda_{1}\to 1}{\lim}-\frac{\left(\lambda_{1}^{-\frac{\alpha }{2}}-\lambda_{1}^{\frac{\beta }{2}}\right)}{\left(\lambda_{1}^{\alpha }-\lambda_{1}^{-\beta }\right)}=\frac{1}{2}$$

This may be considered the initial Poisson’s ratio, which corresponds with the limit value of the Poisson’s ratio for incompressible solids within the linear theory of elasticity. It must be noted that both tangent Young’s modulus and Poisson’s ratio will not be constant in general; their values will change as the deformation continues.

### 5.2. Simple Shear

In cases of simple shear, we have:

$$\{\begin{array}{l} x_{1}=X_{1}+\gamma X_{2}\\ x_{2}=X_{2}\\ x_{3}=X_{3} \end{array}\Rightarrow \;F=\left[\begin{array}{ccc} 1 & \gamma & 0\\ 0 & 1 & 0\\ 0 & 0 & 1 \end{array}\right],\;\;C=F^{T}F=\left[\begin{array}{ccc} 1 & \gamma & 0\\ & \gamma^{2}+1 & 0\\ \mathrm{sym} & & 1 \end{array}\right]$$

where C is the material deformation tensor and $\gamma =\frac{\Delta u}{h}>0$, while Δu is the relative displacement of sheared layers, which are at a distance h from one-another. Polar decomposition of material deformation gradient gives us:

(27) $$\;\;R=\left[\begin{array}{ccc} \cos\left(\frac{\pi }{2}-2\phi \right) & -\sin\left(\frac{\pi }{2}-2\phi \right) & 0\\ \sin\left(\frac{\pi }{2}-2\phi \right) & \cos\left(\frac{\pi }{2}-2\phi \right) & 0\\ 0 & 0 & 1 \end{array}\right]$$

(28) $$\;\;U=\left[\begin{array}{ccc} \frac{2}{\sqrt{4+\gamma^{2}}} & \frac{\gamma }{\sqrt{4+\gamma^{2}}} & 0\\ & \frac{2+\gamma^{2}}{\sqrt{4+\gamma^{2}}} & 0\\ \mathrm{sym} & & 1 \end{array}\right]=\frac{1}{1+\lambda_{1}^{2}}\left[\begin{array}{ccc} 2\;\lambda_{1} & \lambda_{1}^{2}-1 & 0\\ & \frac{\lambda_{1}^{4}+1}{\lambda_{1}} & 0\\ \mathrm{sym} & & 1+\lambda_{1}^{2} \end{array}\right]$$

where

$$\mathrm{tg}\phi =\frac{2}{\sqrt{\gamma^{2}+4}-\gamma }\;\;\;\Leftrightarrow \;\;\;\gamma =\mathrm{tg}\phi -\frac{1}{\mathrm{tg}\phi }\;\;,\;\;\gamma \in \left(\frac{\pi }{4};\frac{\pi }{2}\right)$$

and ϕ is the angle between the direction of shearing (axis $x_{1}$) and the direction of maximum principal stretch $\lambda_{1}$. Principal stretches are equal:

(29) $$\lambda_{1}=\sqrt{C_{1}}=\sqrt{1+\frac{\gamma }{2}\left(\gamma +\sqrt{\gamma^{2}+4}\right)}=\frac{1}{\lambda_{2}}\in \left[1\;;+\infty \right)$$

(30) $$\lambda_{2}=\sqrt{C_{2}}=\sqrt{1+\frac{\gamma }{2}\left(\gamma -\sqrt{\gamma^{2}+4}\right)}=\frac{1}{\lambda_{1}}\in \left(0\;;1\right]$$

(31) $$\lambda_{3}=\sqrt{C_{3}}=1$$

This is an isochoric deformation: $J=\frac{dV}{dV_{R}}=\lambda_{1}\lambda_{2}\lambda_{3}=1$, thus no distinction between compressible and incompressible solids is made. Magnitude of shear deformation as well as orientation of principal stretches may be expressed in terms of principal stretches:

(32) $$\gamma =\sqrt{\lambda_{1}^{2}+\frac{1}{\lambda_{1}^{2}}-2}=\sqrt{\lambda_{2}^{2}+\frac{1}{\lambda_{2}^{2}}-2}\;\;,\;\;\;\;\;\mathrm{tg}\phi =\lambda_{1}$$

**Figure 1:**
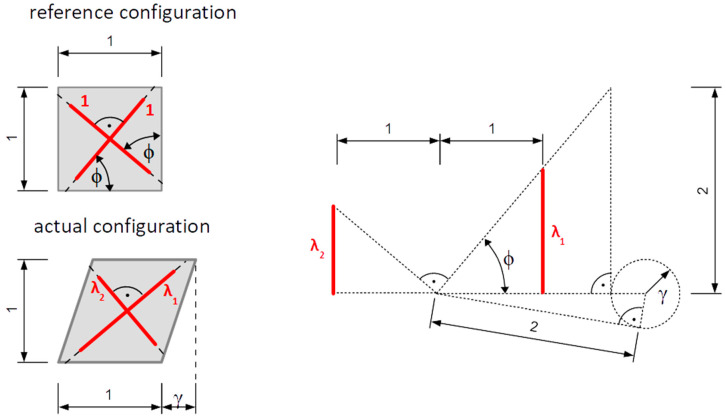
Geometrical interpretation of parameters describing the simple shear state.

Graphical interpretation of the introduced quantities is presented in Figure 1.

We shall now assume that the constitutive law governing the behavior of the material is such that the stress tensors $T^{\left(\alpha ,\beta \right)}$ and strain tensors $E^{\left(\alpha ,\beta \right)}$ are coaxial. In such a case, the Kirchhoff–de Saint-Venant elastic potential may be assumed for a chosen pair of those tensors. Let us assume that in the basis of eigenvectors of $E^{\left(\alpha ,\beta \right)}$, we have:

(33) $$E^{\left(\alpha ,\beta \right)}=\frac{1}{\alpha +\beta }\left[\begin{array}{ccc} \left(\lambda_{1}^{\alpha }-\lambda_{1}^{-\beta }\right) & 0 & 0\\ & \left(\lambda_{1}^{-\alpha }-\lambda_{1}^{\beta }\right) & 0\\ \mathrm{sym} & & 0 \end{array}\right]$$

(34) $$T_{S}=\left[\frac{P\left(\lambda_{1}\right)}{A_{0}}\right]\left[\begin{array}{ccc} {\tilde{S}}_{1}\; & 0 & 0\\ & {\tilde{S}}_{2} & 0\\ \mathrm{sym} & & 0 \end{array}\right]$$

An assumption of coaxiality of those tensors is, in fact, an assumption on the material’s physical characteristics, constraining constitutive modelling in some way. Then, in the original basis:

(35) $$T_{S}=\left[\frac{P\left(\lambda_{1}\right)}{A_{0}}\right]\left[\begin{array}{ccc} \frac{{\tilde{S}}_{1}+\lambda_{1}^{2}{\tilde{S}}_{2}}{1+\lambda_{1}^{2}} & \left({\tilde{S}}_{1}-{\tilde{S}}_{2}\right)\frac{\lambda_{1}}{1+\lambda_{1}^{2}} & 0\\ & \frac{\lambda_{1}^{2}{\tilde{S}}_{1}+{\tilde{S}}_{2}}{1+\lambda_{1}^{2}} & 0\\ \mathrm{sym} & & 0 \end{array}\right]$$

We may determine the Piola–Kirchhoff stress tensor of the first kind on the original basis:

(36) $$T_{R}=F\;T_{S}=\left[\frac{P\left(\lambda_{1}\right)}{A_{0}}\right]\left[\begin{array}{ccc} \frac{\lambda_{1}^{2}{\tilde{S}}_{1}+{\tilde{S}}_{2}}{1+\lambda_{1}^{2}}\; & \frac{\lambda_{1}^{4}{\tilde{S}}_{1}-{\tilde{S}}_{2}}{\lambda_{1}\left(1+\lambda_{1}^{2}\right)} & 0\\ \left({\tilde{S}}_{1}-{\tilde{S}}_{2}\right)\frac{\lambda_{1}}{1+\lambda_{1}^{2}} & \frac{\lambda_{1}^{2}{\tilde{S}}_{1}+{\tilde{S}}_{2}}{1+\lambda_{1}^{2}} & 0\\ 0 & 0 & 0 \end{array}\right]$$

The $T_{12}$ component of $T_{R}$ is interpreted as a nominal shear stress parallel to $x_{1}$ applied to a plane perpendicular to $x_{2}$. For this reason, we shall transcribe:

(37) $$\frac{\lambda_{1}^{4}{\tilde{S}}_{1}-{\tilde{S}}_{2}}{\lambda_{1}\left(1+\lambda_{1}^{2}\right)}=1\;\;\;\;\;\;\Rightarrow \;\;\;\;\;\;{\tilde{S}}_{2}=\lambda_{1}^{4}{\tilde{S}}_{1}-\lambda_{1}\left(1+\lambda_{1}^{2}\right)$$

The use of Equation (8) enables us to express the components of any stress tensor $T^{\left(\alpha ,\beta \right)}$ in terms of material stresses and principal stretches. Considering Equation (37), we may express the only non-zero components as follows:

(38) $$T_{11}^{\left(\alpha ,\beta \right)}=\left[\frac{P\left(\lambda_{1}\right)}{A_{0}}\right]\lambda_{1}\frac{\left(\alpha +\beta \right)\;}{\alpha \lambda_{1}^{\alpha -1}+\beta \lambda_{1}^{-\beta -1}}{\tilde{S}}_{1}$$

(39) $$T_{22}^{\left(\alpha ,\beta \right)}=\left[\frac{P\left(\lambda_{1}\right)}{A_{0}}\right]\frac{\left(\alpha +\beta \right)\;}{\alpha \lambda_{1}^{1-\alpha }+\beta \lambda_{1}^{1+\beta }}\left[\lambda_{1}^{3}{\tilde{S}}_{1}-\left(1+\lambda_{1}^{2}\right)\right]$$

We shall now assume a linear constitutive relationship between $T^{\left(\alpha ,\beta \right)}$ and $E^{\left(\alpha ,\beta \right)}$ in the form of Equation (15), corresponding with the Kirchhoff–de Saint-Venant potential:

(40) $$T_{11}^{\left(\alpha ,\beta \right)}=2\mu E_{11}^{\left(\alpha ,\beta \right)}+\Lambda \left(E_{11}^{\left(\alpha ,\beta \right)}+E_{22}^{\left(\alpha ,\beta \right)}+E_{33}^{\left(\alpha ,\beta \right)}\right)$$

(41) $$T_{22}^{\left(\alpha ,\beta \right)}=2\mu E_{22}^{\left(\alpha ,\beta \right)}+\Lambda \left(E_{11}^{\left(\alpha ,\beta \right)}+E_{22}^{\left(\alpha ,\beta \right)}+E_{33}^{\left(\alpha ,\beta \right)}\right)$$

(42) $$T_{33}^{\left(\alpha ,\beta \right)}=2\mu E_{33}^{\left(\alpha ,\beta \right)}+\Lambda \left(E_{11}^{\left(\alpha ,\beta \right)}+E_{22}^{\left(\alpha ,\beta \right)}+E_{33}^{\left(\alpha ,\beta \right)}\right)=0$$

In the case of simple shear $E_{33}^{\left(\alpha ,\beta \right)}=0$, the last equation will only be satisfied if

$$\mathrm{tr}\left(E^{\left(\alpha ,\beta \right)}\right)=\frac{\left(\lambda_{1}^{a}+\lambda_{1}^{-\alpha }\right)-\left(\lambda_{1}^{\beta }+\lambda_{1}^{-\beta }\right)}{\alpha +\beta }=0$$

This must hold true for any value of $\lambda_{1}$; therefore, it is possible only if α=β, which is what was assumed in [15], unless $S_{3}\neq 0$. In the latter case, however, the true stress $\sigma_{33}=S_{3}\neq 0$. If α=β, then both stress and strain state are plane; however, if α≠β, then the strain state is plane and the stress state is anti-plane. This is not a situation which is commonly assumed in basic theoretical analyses of sheared elements [21], although it should not be disregarded. In fact, in the case of a lap joint in which a thin layer of flexible adhesive connects two elements of much higher stiffness, the case of plane strain states in adhesives is approximately true in the regions which are sufficiently distant from free edge side boundaries of the adhesive. It must be noted that the distribution of stress and strain is then no longer uniform—it is approximately constant only within the interior and far from boundaries. It may emerge that assuming α≠β may provide a sufficiently precise engineering estimate of the behavior of such a system, even if uniform distribution of stress is assumed. Thus, we shall not disregard the case of anti-plane stress state in advance. The transverse normal stress is then calculated according to Equation (42). The rest of the equations enable us to express the nominal shear stress in terms of maximum principal stretch, parameters α,β, and material constants only. Equation (40) gives us:

(43) $$\left[\frac{P\left(\lambda_{1}\right)}{A_{0}}\right]{\tilde{S}}_{1}=\frac{\alpha \lambda_{1}^{\alpha -1}+\beta \lambda_{1}^{-\beta -1}}{\lambda_{1}{\left(\alpha +\beta \right)}^{2}}\left\{2\mu \left(\lambda_{1}^{\alpha }-\lambda_{1}^{-\beta }\right)+\Lambda \left[\left(\lambda_{1}^{\alpha }-\lambda_{1}^{-\beta }\right)+\left(\lambda_{1}^{-\alpha }-\lambda_{1}^{\beta }\right)\right]\right\}$$

Substituting it into Equation (39):

(44) $$\begin{array}{c} T_{22}^{\left(\alpha ,\beta \right)}=\frac{\lambda_{1}^{2}}{\left(\alpha +\beta \right)}\frac{\;\alpha \lambda_{1}^{\alpha -1}+\beta \lambda_{1}^{-\beta -1}}{\alpha \lambda_{1}^{1-\alpha }+\beta \lambda_{1}^{1+\beta }}\left\{2\mu \left(\lambda_{1}^{\alpha }-\lambda_{1}^{-\beta }\right)+\Lambda \left[\left(\lambda_{1}^{\alpha }-\lambda_{1}^{-\beta }\right)+\left(\lambda_{1}^{-\alpha }-\lambda_{1}^{\beta }\right)\right]\right\}\\ -\left[\frac{P\left(\lambda_{1}\right)}{A_{0}}\right]\frac{\left(\alpha +\beta \right)\left(1+\lambda_{1}^{2}\right)\;}{\alpha \lambda_{1}^{1-\alpha }+\beta \lambda_{1}^{1+\beta }} \end{array}$$

This may be finally substituted into Equation (41):

(45) $$\left[\frac{P\left(\lambda_{1}\right)}{A_{0}}\right]=\frac{\lambda_{1}^{2}}{\left(1+\lambda_{1}^{2}\right)}\frac{\;\alpha \lambda_{1}^{\alpha -1}+\beta \lambda_{1}^{-\beta -1}}{{\left(\alpha +\beta \right)}^{2}}\left\{2\mu \left(\lambda_{1}^{\alpha }-\lambda_{1}^{-\beta }\right)+\Lambda \left[\left(\lambda_{1}^{\alpha }-\lambda_{1}^{-\beta }\right)+\left(\lambda_{1}^{-\alpha }-\lambda_{1}^{\beta }\right)\right]\right\}\;-\frac{1}{\left(1+\lambda_{1}^{2}\right)}\frac{\alpha \lambda_{1}^{1-\alpha }+\beta \lambda_{1}^{1+\beta }}{{\left(\alpha +\beta \right)}^{2}}\left\{2\mu \left(\lambda_{1}^{-\alpha }-\lambda_{1}^{\beta }\right)+\Lambda \left[\left(\lambda_{1}^{\alpha }-\lambda_{1}^{-\beta }\right)+\left(\lambda_{1}^{-\alpha }-\lambda_{1}^{\beta }\right)\right]\right\}$$

If α=β is assumed, then:

(46) $$\left[\frac{P\left(\lambda_{1}\right)}{A_{0}}\right]=\mu \frac{\;\lambda_{1}^{2\alpha +1}-\lambda_{1}^{1-2\alpha }}{\alpha \left(1+\lambda_{1}^{2}\right)}$$

We can now find an interpretation of elastic constants in the assumed potential. An engineering shear strain is calculated as a component of a small strain tensor:

$$\varepsilon =\frac{1}{2}\left[\left(F-I\right)+{\left(F-I\right)}^{T}\right]=\left[\begin{array}{ccc} 0\; & \frac{\gamma }{2} & 0\\ & 0 & 0\\ \mathrm{sym} & & 0 \end{array}\right]$$

The initial tangent Kirchhoff’s modulus is calculated as follows:

(47) $$G_{t,0}=\frac{1}{2}\frac{d}{d\varepsilon_{12}}{\left[\frac{P\left(\lambda_{1}\right)}{A_{0}}\right]|}_{\varepsilon_{12}=0}=\frac{d}{d\gamma }{\left[\frac{P\left(\lambda_{1}\right)}{A_{0}}\right]|}_{\gamma =0}=\mu$$

A material considered physically non-linear may be thus characterized by two elastic constants, μ,Λ , which, in turn, may be expressed in terms of initial tangent Young’s modulus and Kirchhoff’s modulus interpreted in terms of engineering strain and nominal stress, for example, according to Equations (26) and (47):

(48) $$\mu =G_{t,0},\;\;\Lambda =\frac{G_{t,0}\left(E_{t,0}-2G_{t,0}\right)}{3G_{t,0}-E_{t,0}}$$

However, such a solution requires finding such parameters 

α, β for which the stress–strain relationship predicted by the model based on the elastic potential of Kirchhoff–de Saint-Venant is in good agreement with experimental data.

## 6. Released Constraints on Admissible Values of Exponents α,β in a Limit Range of Variation of Stretch

It was mentioned that parameters α,β must be such that either their product is positive or both of them must tend in their limit to 0 in order to satisfy the requirement of infinite measures of strain in limited cases of deformation. These requirements, however, are not met in the case of the Seth–Hill strain tensors, which in fact does not narrow the scope of application of those measures. It was also observed that good agreement between theoretical prediction and experimental results was obtained for α,β, such that αβ<0 or (αβ=0∧α≠β), at least in a certain limited range of variation of stretch [17]. The key property of a generalized measure of strain is that it should provide a one-to-one relationship between stretch and strain. The constraints on admissible values of α,β may be thus released as long as Equation (4) is strictly increasing, which results in its bijectivity. Equation (4) is defined in such a way that for λ=1 it is always equal 0, and its derivative is equal to 1; therefore, it imposes a requirement that within a fixed interval $\left(\lambda_{min};\lambda_{max}\right)$ no stationary point can occur. If α=0, then f(λ) is a rational function with no stationary points. Similarly, if β=0, then f(λ) is a power function with no stationary points. If we reject the case of α=β=0, the stationary point can be found as follows:

(49) $${\frac{df}{d\lambda }|}_{\lambda_{0}}=\frac{1}{\alpha +\beta }\left(\alpha \lambda_{0}^{\alpha -1}+\beta \lambda_{0}^{-\beta -1}\right)=0\;\;\;\Rightarrow \;\;\;\;\lambda_{0}={\left[-\frac{\beta }{\alpha }\right]}^{\frac{1}{\alpha +\beta }}$$

We next required that:

(50) $$\left({\left[-\frac{\beta }{\alpha }\right]}^{\frac{1}{\alpha +\beta }}\leq \;\lambda_{min}\right)\vee \left({\left[-\frac{\beta }{\alpha }\right]}^{\frac{1}{\alpha +\beta }}\geq \;\lambda_{max}\right)$$

The above inequalities constitute the domain for the data fitting algorithms used in order to determine optimal parameters α, β in the models mentioned above. 

## 7. Experimental Verification of Proposed Models

The proposed constitutive model was verified with the experimental results of the uniaxial tension test as well as the simple shear test for the SikaPM-type polyurethane-based resin (Cracow, Sika Poland). An important property of this material is the effect of strain rate (visco-elasticity). Having this in mind, we should emphasize that the presented approach, namely, constitutive modelling with the use of a model of purely elastic material, is valid from the strict theoretical point of view only in those cases in which the strain rate is constant. The parameters of such a model may be used only for strain rates for which they were determined. It should be noted that in the case of a uniaxial tension test with constant strain rate, a constant stretch rate was achieved. In a shear test, on the other hand, stretch rate was not constant for the constant shear angle rate. This can be seen from Equation (32), which shows the non-linear relationship between shear angle and stretch, as well as the relationship between shear angle rate and stretch rate, given by (51):

(51) $$\dot{\gamma }=\frac{\lambda^{2}-1}{2\;\lambda^{\frac{3}{2}}\;\sqrt{\lambda^{2}-2\lambda +1}}\;\dot{\lambda }$$

Although this relationship makes it necessary to account for viscous properties of the material in its modelling, this incompatibility may, to some extent, be surpassed by using very low stretch rates. In such cases, the effect of viscosity is of lesser importance and may even be neglected in some situations.

### 7.1. Uniaxial Tension

**Figure 2:**
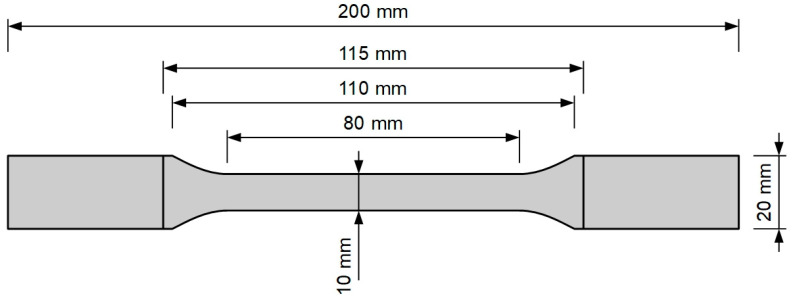
Dimensions of a dogbone specimen.

**Figure 3:**
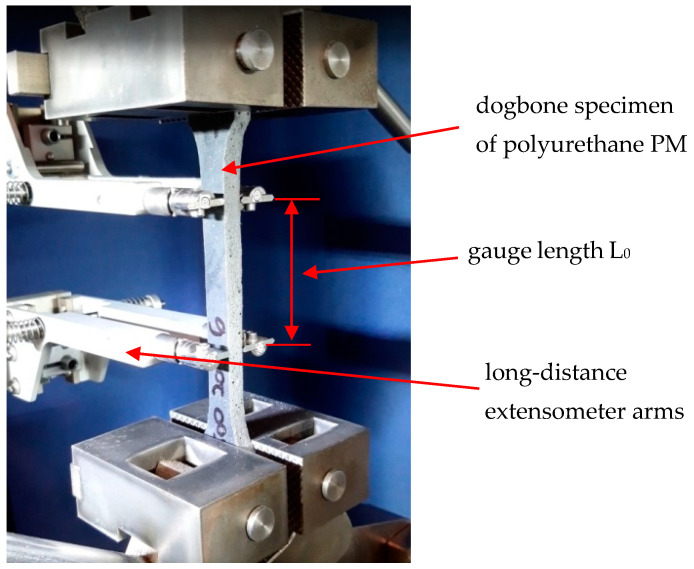
Uniaxial tension test of a dogbone specimen.

**Figure 4:**
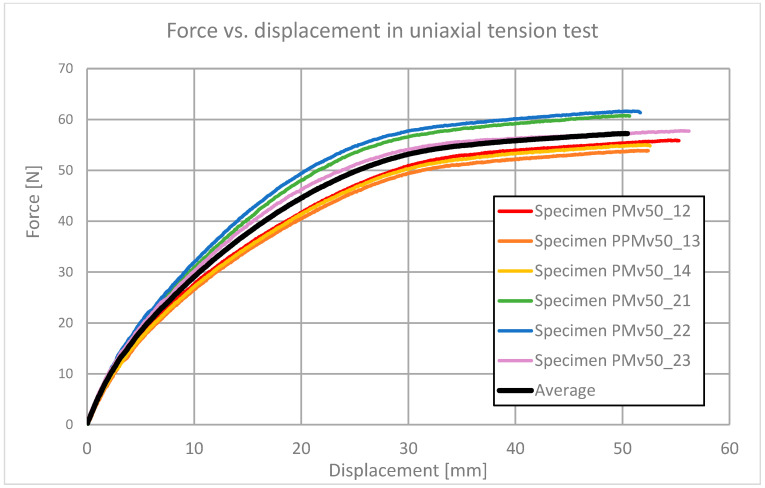
The force–displacement curve for six dogbone specimens in uniaxial tension tests, and an average curve.

The uniaxial tension test was performed [22] with the use of dogbone specimens (Figure 2) on a Zwick/Roell 1455 testing machine with maximum force of 20 kN and force and extensometer precision of 0.1 kN and 0.005 mm, respectively. The strain rate was 100%/min, and the gauge length of the extensometer was 50 mm. Six specimens were tested (see Figure 3), and the results are shown in Figure 4. The average force–displacement relationship was determined and used for verification of the theoretical model.

#### Calibration of Parameters α and β

**Figure 5:**
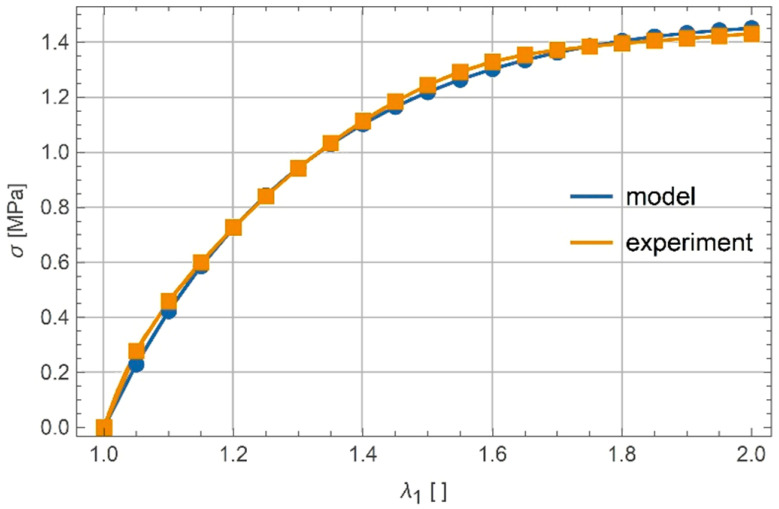
Experimental and calibrated model response for uniaxial tension.

The elastic modulus of the material E was determined according to the initial slope of the uniaxial response and was equal to 5 MPa. Virtually incompressible material was assumed (ν=0.499). The only remaining material parameters in the model presented in Equation (25) were α and β. They were obtained by the calibration of model response with an average experimental response of six samples. The actual calibration was performed in Mathematica [23] by minimizing differences of the model and the measured response. The calibration gives $\alpha =-8.443\cdot 10^{-2}$ and $\beta =8.457\cdot 10^{-2}$. The results are shown in Figure 5.

### 7.2. Shear 

**Figure 6:**
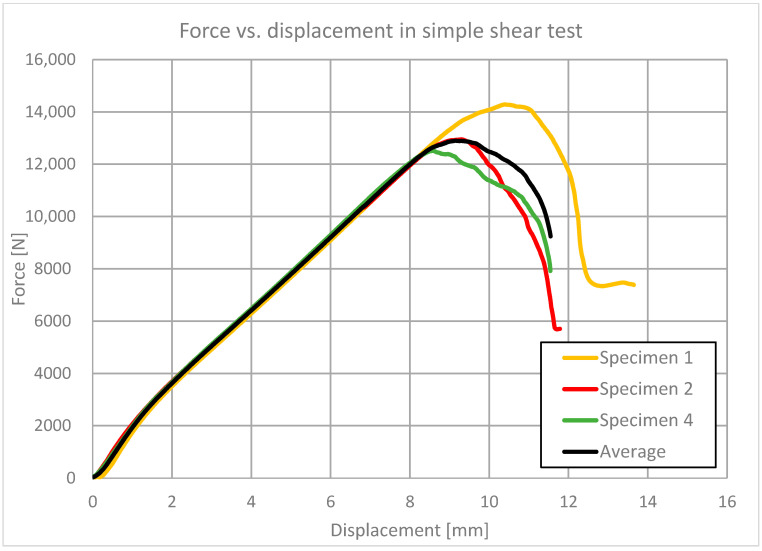
The force–displacement curve for four 100 × 100 × 10 three-concrete block specimens in simple shear tests, and an average curve.

The simple shear test was performed [22] as a triplet test on concrete blocks. The testing machine was a Z100 Zwick/Roell with maximum force of 100 kN and force and extensometer precision of 1 kN and 0.01 mm, respectively. The sheared area dimensions were 100 × 100 mm, and the thickness of the sheared layer of PM resin was 10 mm. The shear angle rate was 100%/min. Three specimens were tested. The results are shown in Figure 6. Average values of the force–displacement relationship were determined and used for verification of the theoretical model. 

#### Calibration of Parameters α and β

**Figure 7:**
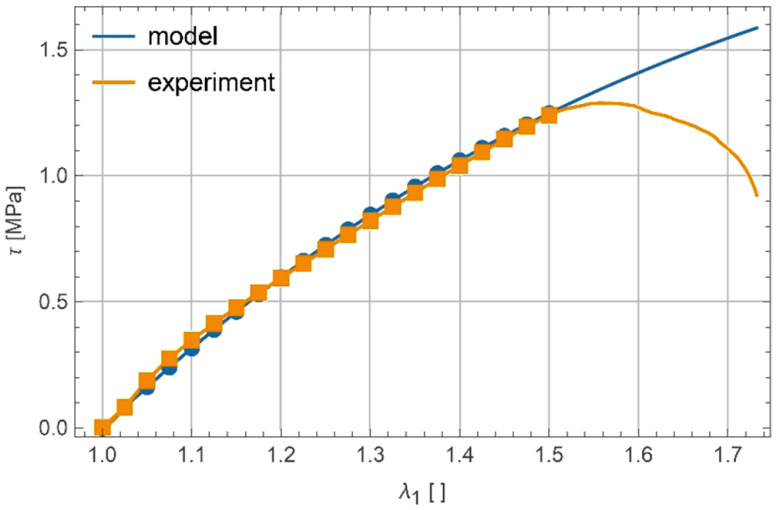
Experimental and calibrated model response for shear.

Elastic modulus and Poisson’s ratio were assumed to be same as in the uniaxial test, and such parameters α and β were sought that gave the smallest difference between the model predictions and the experimental results. The procedure of calibration was the same as for uniaxial tension and gave $\alpha =1.665\times 10^{-5}$ and $\beta =1.735\times 10^{-5}$. The response of the calibrated model is compared to the experiment in Figure 7. Discrepancies between the experimental results and results predicted by the theoretical model for $\lambda_{1}>1.5$ are due to the fact that calibration process concerned only a truncated range of $\lambda_{1}\in \left(0;1.5\right)$, because it can be observed that for larger values of principal stretch, failure mechanisms become dominant in the deformation of the sample. Such a rapid change in the deformation mechanism surely cannot be properly modelled with the use of the considered model based on a single elastic potential.

### 7.3. Discussion

**Figure 8:**
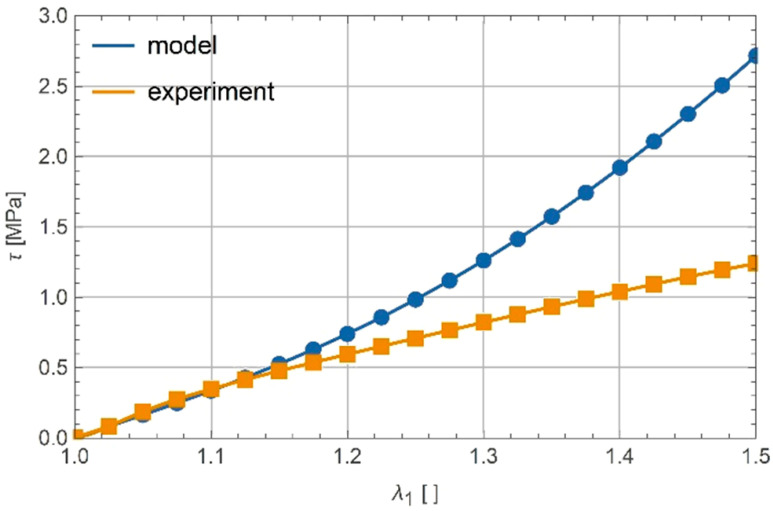
Response in shear with α and β calibrated for tension.

Parameters α and β that were obtained by calibration to experiments were not the same for uniaxial tension and pure shear. Furthermore, if α and β obtained from calibration of tensile response were used to model shear response, the match was very poor (see Figure 8).

**Figure 9:**
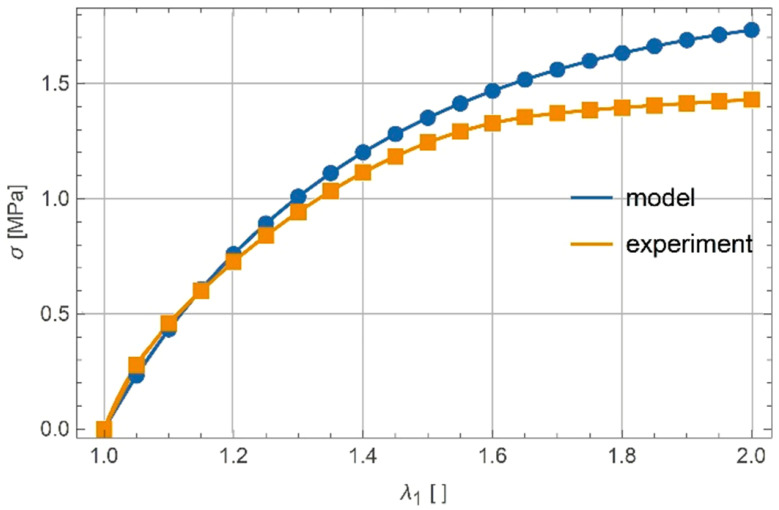
Response in tension with α and β calibrated for shear.

Similarly, the model gave a poor prediction if α and β obtained from the calibration of shear response were used to model tensile response (see Figure 9).

It can thus be concluded that no such set of parameters α and β exist that would provide a satisfying estimate of the experimental results with the use of theoretically derived formulae given by Equations (25) and (45) within the considered range of strain. Among many possible explanations of such a situation, we may specify the following:

- The true stress and strain state in the samples differed considerably from what was assumed in the theoretical derivations. This especially concerns the simple shear test in which the stress and strain distribution in the sheared layer is strongly influenced by boundary effects;
- In the case of very large strains, it must be stated that the physical mechanisms of deformation become qualitatively distinct from those which are dominant in the domain of small strain—in particular, breaking the polymer chains becomes more significant. As a result, any approximation of the material’s characteristics with the use of only a single constitutive model—which was assumed to be valid within the whole range of strains—should be expected to fail if the strain becomes sufficiently large;
- The use of the Kirchhoff–de Saint-Venant elastic potential may be inappropriate for the description of the considered material, due to viscous properties of the considered material.

Concerning the latter, it must be noted that within the range of principal stretch in simple shear λ ∈ (1;1,5) the respective stretch rate $\dot{\lambda }$ varied less than ±50%. Simple tension tests on PM dogbone specimens corresponding with shear rates 10%/min, 100%/min, and 1000%/min indicated that within the mentioned range of strain rates, the initial tangent Young’s modulus varied less than 15%. This suggests that viscosity is of lesser importance in the considered cases. The mentioned discrepancies also concern relatively small deformations; therefore, it may be suspected that the second of the possible causes mentioned above is also not the dominant one, and it is in fact a poor realization of the simple shear state that makes it impossible to find a single set of values of α and β that would enable proper description of the material’s behavior in both simple tension and simple shear.

The proposed approach may provide us with a simple computational model approximating non-linear characteristics of the material with either Equation (25) in the case of simple tension, or Equation (45) in the case of simple shear for appropriate values of α and β. Those parameters of the measure of strain should not be considered the same for those two distinct mechanical states. Such modelling makes no account for viscosity; therefore, the proposed approach is valid only in the case when strain rate is constant (or varies to a small extent) and if the strain itself is sufficiently small—then, the determined values of α and β would correspond only with an assumed strain rate. The presented approach can by no means be considered constitutive modelling; however, it still may provide a simple tool for the description of materials of non-linear characteristics in the design process. For this reason, the use of the Kirchhoff–de Saint-Venant elastic potential for energy conjugate measures of strain and stress by Darijani and Naghdabadi corresponding with α and β determined independently for simple tension and for simple shear can be considered a “design model” rather than a “constitutive” model. Such a “design model” provides designers with simple, general formulas describing the response of materials with non-linear characteristics. Those formulae generally depend on four parameters, i.e., $E_{t,0},G_{t,0},\alpha \;\mathrm{and}\;\beta$, which enables calibration of the force–displacement curve with a great variety of experimental results.

## 8. Summary and Conclusions

Modelling of materials exhibiting non-linear physical characteristics was discussed in detail. Explicit relationships between nominal stress and stretch were presented in cases of simple tension and simple shear with the use of nonlinear strain measures by Darijani and Naghdabadi and a linear constitutive relationship corresponding with the Kirchhoff–de Saint-Venant elastic potential. Constant parameters of the assumed potential were interpreted in terms of initial tangent elastic constants. The proposed model was verified with experimental data. Simple tension tests were performed with the use of dogbone specimens and simple shear tests were performed with the use of triplet concrete block specimens. The deformation rate was constant in order to minimize the influence of viscous properties of the adhesive. Parameters of the strain measure α and β were optimized in order to ensure that the derived theoretical estimates corresponded with the experimental data well within the assumed range of values of principal stretch. It emerged that optimal values of α and β were different for simple tension and for simple shear—optimal values of α and β determined for simple tension state provided very poor agreement with the experimental results of the simple shear test and vice versa. Based on the results of simple tension tests corresponding with various stretch rates, the influence of viscosity was estimated to be small. Due to relatively large calibration capabilities of the presented four-parameter model, it was concluded that observed discrepancies in the description of material in both tension and shear with a single set of parameters were due to poor experimental realization of the simple shear state. The presented approach may still find application as a practical computational design model describing materials of non-linear characteristics in simple mechanical states, namely, in the process of designing composite connectors, lap joints, etc., with the use of flexible adhesives.

## Data Availability

The data presented in this study may be made available on request from the corresponding author. The data are not publicly available due to technical and legal reasons.
